# Bilateral Gustatory Disturbance Associated With Unilateral Putaminal Hemorrhage: A Case Report and Literature Review

**DOI:** 10.7759/cureus.110644

**Published:** 2026-06-11

**Authors:** Kohei Hashida, Tatsuya Tanaka, Junpei Kato, Takao Zama, Akira Matsuno

**Affiliations:** 1 Department of Neurosurgery, International University of Health and Welfare Narita Hospital, Narita, JPN

**Keywords:** bilateral ageusia, case report, central gustatory pathway, gustatory disturbance, intracerebral hemorrhage, literature review, putaminal hemorrhage

## Abstract

Taste disturbance is relatively common after acute stroke but is often overlooked because it is subjective and frequently overshadowed by more prominent neurological deficits. Gustatory dysfunction specifically associated with putaminal hemorrhage has rarely been reported. We describe a 43-year-old right-handed woman who developed acute bilateral loss of taste in close temporal association with a left putaminal hemorrhage, despite clinically preserved olfaction and only mild sensory symptoms. Brain magnetic resonance imaging demonstrated a small left putaminal hemorrhage with surrounding edema extending toward the thalamic region. Electrogustometry performed on hospital day 11, after partial subjective improvement, revealed bilateral but asymmetric gustatory impairment. Alternative causes, including peripheral cranial nerve dysfunction, medication-induced dysgeusia, postinfectious etiologies, and metabolic abnormalities, were considered less likely based on the clinical course and diagnostic evaluation. Her taste disturbance gradually improved and was completely resolved within two months. In this patient, the temporal association and imaging findings suggest, but do not prove, transient dysfunction of adjacent subcortical gustatory pathways, including thalamocortical projections or fibers near the internal capsule. Gustatory disturbance after putaminal hemorrhage is rarely reported and may be underrecognized in routine stroke assessment. Targeted inquiry regarding taste perception and, when available, objective gustatory testing may improve detection and characterization of this subtle manifestation in patients with deep hemispheric stroke.

## Introduction

Taste disturbance after stroke is relatively common but frequently overlooked in clinical practice. Prospective studies have reported gustatory dysfunction in approximately 30% of patients with acute stroke; however, this symptom is often underdocumented because it is subjective and frequently overshadowed by more prominent neurological deficits [1].

Central gustatory pathways involve the brainstem, thalamus, insula, frontal operculum, and adjacent subcortical projection fibers [2-7]. Therefore, stroke lesions at various levels may cause hemiageusia, bilateral taste disturbance, or dysgeusia [2-7]. The insula and frontal operculum are regarded as primary cortical gustatory regions, whereas putaminal or peri-putaminal lesions may affect adjacent subcortical gustatory projection pathways rather than the primary gustatory cortex itself. However, gustatory disturbance associated with putaminal hemorrhage has rarely been reported, and its clinical features, laterality patterns, and prognosis remain insufficiently characterized [2,5,6].

Herein, we report a case of bilateral gustatory disturbance associated with unilateral putaminal hemorrhage and review previously reported cases of gustatory disturbance associated with putaminal lesions.

## Case presentation

A 43-year-old right-handed woman with a history of hypertension, stage 4 chronic kidney disease, and hyperuricemia presented to the emergency department with acute sensory symptoms and taste loss. She was an active smoker, consuming approximately 20 cigarettes per day using both electronic and conventional cigarettes, and reported regular alcohol intake of approximately 56 g/day. Her preadmission medications included spherical carbon adsorbent 2,000 mg three times daily, topiroxostat 40 mg twice daily, and irbesartan 100 mg once daily. No new medication had been started immediately before the onset of taste disturbance. She had no recent history of upper respiratory tract infection, COVID-19 infection, fever, oral infection, dental procedure, or oral pain.

On the morning of presentation, she developed sensory disturbance in the right hand and vomiting. During lunch on the same day, while eating instant noodles, she noticed complete loss of taste perception despite preserved olfaction. Because these symptoms persisted, she visited the emergency department later that evening.

On arrival, she was fully alert, with a Glasgow Coma Scale score of E4V5M6. Her blood pressure was 188/124 mmHg, heart rate was 110 beats/min, respiratory rate was 15 breaths/min, oxygen saturation was 98% on room air, and body temperature was 37.3°C. Neurological examination revealed sensory disturbance and numbness in the right forearm and palm. A mild right-sided Barré sign was present, whereas the Mingazzini test was negative. There was no facial palsy, aphasia, dysarthria, dysphagia, or grip weakness. Cranial nerve and oral examinations showed no peripheral facial, glossopharyngeal, vagal, or oral mucosal abnormalities. Olfaction was clinically reported as preserved by the patient, although formal olfactory testing was not performed. Taste sensation was subjectively absent. The National Institutes of Health Stroke Scale score was 1 [8].

**Figure 1:**
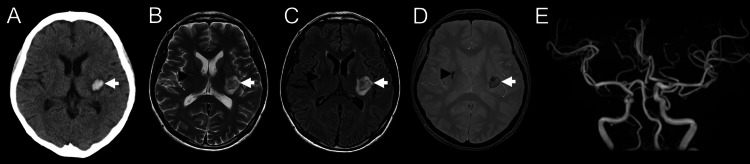
Neuroimaging findings of putaminal hemorrhage. (A) Non-contrast head computed tomography on admission showing a small acute hemorrhage in the left putamen, measuring approximately 13 mm in maximum diameter (white arrow). (B, C) Axial T2-weighted and fluid-attenuated inversion recovery magnetic resonance images on hospital day 2 demonstrating the left putaminal hemorrhage with surrounding edema extending toward the thalamic region (white arrows). This peri-putaminal lesion was located near the internal capsule and thalamocortical projection area, where transient involvement of adjacent subcortical gustatory pathways was considered possible. (D) T2*-weighted imaging showing a hypointense lesion corresponding to the acute left putaminal hemorrhage (white arrow) and an old right putaminal hemorrhagic lesion suggestive of a previous hemorrhagic scar (black arrowhead). (E) Magnetic resonance angiography showing no vascular malformation, aneurysm, or major arterial stenosis.

Non-contrast head computed tomography revealed a small acute left putaminal hemorrhage measuring approximately 13 mm in maximum diameter (Figure 1A).

Initial and additional laboratory findings are summarized in Table 1.

**Table 1:** Laboratory findings.

Laboratory test	Result	Reference range
White blood cell count	6.96 × 10³/μL	3.30–8.60 × 10³/μL
Red blood cell count	2.41 × 10⁶/μL	3.86–4.92 × 10⁶/μL
Hemoglobin	8.5 g/dL	11.6–14.8 g/dL
Hematocrit	24.30%	35.1–44.4%
Mean corpuscular volume	100.8 fL	83.6–98.2 fL
Mean corpuscular hemoglobin	35.3 pg	27.5–33.2 pg
Mean corpuscular hemoglobin concentration	35.0 g/dL	31.7–35.3 g/dL
Platelet count	258 × 10³/μL	158–348 × 10³/μL
Prothrombin time-international normalized ratio	0.97	0.90–1.10
Activated partial thromboplastin time	22.9 seconds	25.0–40.0 seconds
Blood urea nitrogen	24.3 mg/dL	8.0–20.0 mg/dL
Serum creatinine	1.74 mg/dL	0.46–0.79 mg/dL
Estimated glomerular filtration rate	26.8 mL/min/1.73 m²	–
Total bilirubin	0.8 mg/dL	0.4–1.5 mg/dL
Direct bilirubin	<0.1 mg/dL	0.0–0.2 mg/dL
C-reactive protein	0.10 mg/dL	0.00–0.14 mg/dL
Aspartate aminotransferase	74 U/L	13–30 U/L
Alanine aminotransferase	25 U/L	7–23 U/L
γ-glutamyl transpeptidase	107 U/L	9–32 U/L
Alkaline phosphatase	76 U/L	38–113 U/L
Lactate dehydrogenase	260 U/L	124–222 U/L
Sodium	140 mmol/L	138–145 mmol/L
Potassium	3.8 mmol/L	3.6–4.8 mmol/L
Chloride	104 mmol/L	101–108 mmol/L
Calcium	8.2 mg/dL	8.8–10.1 mg/dL
Inorganic phosphorus	3.9 mg/dL	2.7–4.6 mg/dL
Blood glucose	134 mg/dL	73–109 mg/dL
Hemoglobin A1c	4.60%	4.9–6.0%
Magnesium	1.3 mg/dL	1.8–2.6 mg/dL
Serum iron	45 μg/dL	40–188 μg/dL
Ferritin	117 ng/mL	5–152 ng/mL
Vitamin B12	284 pg/mL	180–914 pg/mL
Folate	5.4 ng/mL	≥4.0 ng/mL
Serum copper	175 μg/dL	68–128 μg/dL
Serum zinc	55 μg/dL	80–130 μg/dL
Thyroid-stimulating hormone	2.16 μIU/mL	0.50–5.00 μIU/mL
Free triiodothyronine	1.9 pg/mL	2.3–4.0 pg/mL
Free thyroxine	0.9 ng/dL	0.9–1.7 ng/dL
N-terminal pro-brain natriuretic peptide	529 pg/mL	≤125 pg/mL

The results showed macrocytic anemia, stage 4 chronic kidney disease, mild elevation of hepatobiliary enzymes, mild hypocalcemia, and hypomagnesemia. The white blood cell count, platelet count, C-reactive protein level, serum electrolytes other than calcium and magnesium, and coagulation parameters were not suggestive of infection, major electrolyte disturbance, or coagulopathy. Iron and ferritin levels were within the reference ranges. Additional testing showed normal vitamin B12 and folate levels, mildly elevated serum copper, and mildly decreased serum zinc. Overall, there was no evidence of infection, coagulopathy, or a major metabolic abnormality sufficient to explain the acute and complete loss of taste.

Based on the diagnosis of left putaminal hemorrhage, conservative treatment was initiated, including intravenous tranexamic acid and strict blood pressure control targeting a systolic blood pressure of 110-140 mmHg. Oral intake was resumed on hospital day 2. Olfaction remained clinically preserved, but complete loss of taste sensation persisted. No dysphagia, dysarthria, or new neurological deficit was observed. Brain magnetic resonance imaging (MRI) performed on hospital day 2 confirmed the small left putaminal hemorrhage with surrounding edema extending toward the thalamic region (Figure 1B, 1C). The lesion was located near the posterior limb of the internal capsule, without direct involvement of the insular cortex or frontal operculum. Diffusion-weighted imaging did not reveal additional acute ischemic lesions. T2*-weighted imaging demonstrated a hypointense lesion corresponding to the acute left putaminal hemorrhage and an additional hypointense lesion in the right putamen, consistent with a previous hemorrhagic scar (Figure 1D). Magnetic resonance angiography showed no vascular malformation, aneurysm, or major arterial stenosis (Figure 1E).

**Figure 2:**
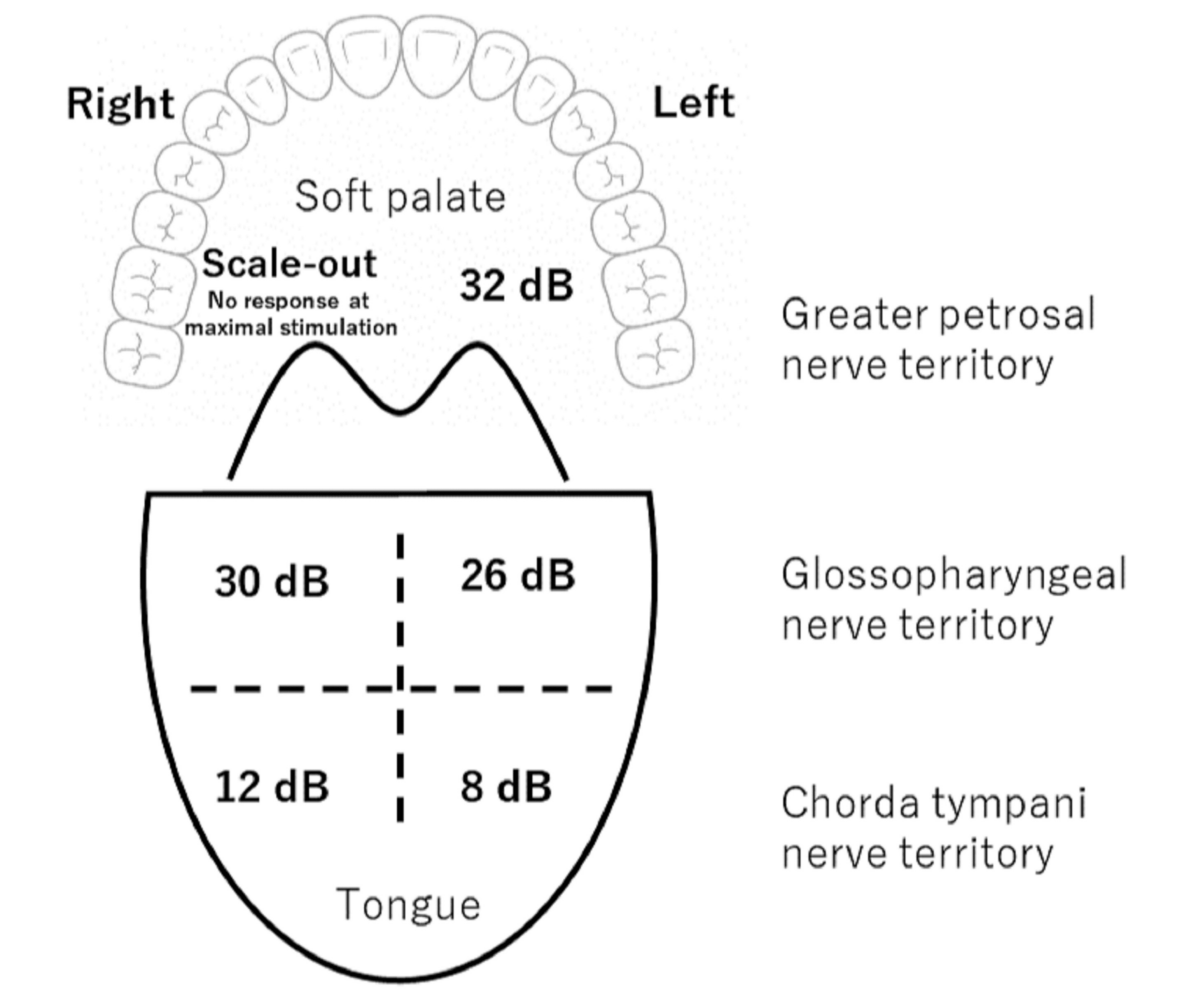
Electrogustometry findings on hospital day 11. The diagram shows taste thresholds in the chorda tympani, glossopharyngeal, and greater petrosal nerve territories. Electrogustometry was performed after partial subjective improvement in taste perception had already begun. Higher thresholds indicate more severe gustatory impairment, and scale-out indicates no response even at maximal stimulation. Although normative thresholds vary according to age, stimulation site, device, and institutional protocol, thresholds in healthy subjects are generally reported to be up to approximately 8 dB at commonly tested sites. The image layout was prepared using Microsoft PowerPoint (Microsoft Corporation, Redmond, WA, USA); no generative artificial intelligence was used.

From hospital day 4, the patient reported gradual subjective improvement in taste perception. On hospital day 11, electrogustometry was performed after partial subjective improvement had already begun; therefore, the examination did not capture the initial complete ageusia (Figure 2).

The bilateral chorda tympani, glossopharyngeal, and greater petrosal nerve territories were tested, and the lowest stimulus intensity at which the patient perceived a taste sensation was recorded as the threshold. Higher thresholds indicate more severe gustatory impairment, and scale-out was defined as no response even at maximal stimulation. In healthy subjects, thresholds are generally reported to be up to approximately 8 dB.

Thresholds in the chorda tympani nerve territory were 12 decibels (dB) on the right and 8 dB on the left, suggesting normal-to-mildly elevated thresholds with right-sided predominance. In the glossopharyngeal nerve territory, thresholds were 30 dB on the right and 26 dB on the left. In the greater petrosal nerve territory, no response was elicited on the right side, even at maximal stimulation, indicating scale-out, whereas the threshold on the left side was 32 dB. These findings supported bilateral but asymmetric gustatory impairment with right-sided predominance. Despite this objective asymmetry, the patient reported minimal subjective awareness of laterality at the time of testing.

Alternative causes of taste disturbance, including zinc deficiency, medication-induced dysgeusia, peripheral cranial nerve dysfunction, postinfectious etiologies, and metabolic abnormalities, could not be definitively excluded. However, these causes were considered less likely because no new medication had been started before symptom onset, there was no recent infectious or oral disease history, olfaction was clinically preserved, and no peripheral cranial nerve deficit was observed. Although mild zinc deficiency may have contributed, it was not considered sufficient to explain the abrupt onset of complete taste loss. Given the temporal association with the left putaminal hemorrhage and the clinical course, a stroke-related central gustatory disturbance was considered the most plausible explanation.

The patient was discharged with a modified Rankin Scale score of 1 [9]. At the two-month outpatient follow-up, she reported complete resolution of taste disturbance without recurrence of neurological symptoms.

## Discussion

Taste disturbance after stroke is not rare, but it is often overlooked in routine clinical practice [1]. Prospective studies have reported gustatory dysfunction in approximately 30% of patients with acute stroke [1]; however, only a small number of reports have specifically described taste disturbance associated with putaminal lesions [2,5,6].

To contextualize the present case, we conducted a targeted narrative review of previously reported cases. PubMed/MEDLINE was searched on March 31, 2026, using the terms “putamen,” “putaminal hemorrhage,” “putaminal infarction,” “taste disturbance,” “gustatory disturbance,” “gustatory dysfunction,” “ageusia,” “hypogeusia,” and “dysgeusia.” English- or Japanese-language articles describing gustatory disturbance associated with putaminal hemorrhage, putaminal infarction, or related putaminal lesions were included. The reference lists of the included articles were also screened. This search identified three publications describing four patients [2,5,6]. Together with the present case, five documented cases were available for comparison. As summarized in Table 2, unilateral putaminal lesions may be associated with variable laterality patterns of gustatory disturbance, including bilateral, ipsilateral, and contralateral impairment [2,5,6]. 

**Table 2:** Reported cases of gustatory disturbance associated with putaminal lesions.

Author (year)	Age/sex	Handedness	Stroke type	Lesion side	Gustatory disturbance	Assessment method	Associated Neurological or Imaging Findings	Outcome
Imamura et al. (2002) [5]	48/M	Left-handed	Hemorrhage	Right putamen	Bilateral severe taste disturbance	Filter-paper disc method	Dysarthria and dysphagia; coexisting multiple infarcts	Persistent deficit
Ito et al. (2006) [6]	39/M	Left-handed	Hemorrhage	Left putamen	Bilateral hypogeusia	Filter-paper disc method	Mild facial palsy and mild hemiparesis	Complete recovery within approximately 3 months
Onoda et al. (2012) [2]	64/M	Not specified	Infarction	Unilateral putamen	Ipsilateral taste disturbance	Electrogustometry and filter-paper disk method	Not detailed	Improved at 2 months
Onoda et al. (2012) [2]	48/M	Not specified	Hemorrhage	Unilateral putamen	Ipsilateral taste disturbance	Electrogustometry and filter-paper disk method	Not detailed	Unknown
Present case	43/F	Right-handed	Hemorrhage	Left putamen	Bilateral taste disturbance, asymmetric on electrogustometry	Electrogustometry	Mild right sensory deficit; perihematomal edema extending toward the thalamic region	Complete recovery at 2 months

In the present case, the patient subjectively experienced bilateral taste loss, whereas electrogustometry demonstrated bilateral but asymmetric impairment. This finding supports the concept that supratentorial gustatory pathways are not strictly unilateral. The central gustatory pathway is generally considered to ascend predominantly ipsilaterally in the lower brainstem, whereas bilateral projections become more relevant above the pontine or midbrain level, involving the thalamus and cortical gustatory areas [2,3]. Therefore, unilateral supratentorial lesions near the putamen, internal capsule, or thalamocortical projections may produce bilateral or variable gustatory symptoms [2,3,5,6].

The precise mechanism of gustatory disturbance in putaminal hemorrhage remains uncertain. The putamen itself is not generally regarded as a primary gustatory center. This view is supported by an activation likelihood estimation meta-analysis of human gustatory neuroimaging studies, which identified the insula and opercular regions as key cortical areas involved in gustatory processing [7]. In the present case, MRI showed a small left putaminal hemorrhage with surrounding edema extending toward the thalamic region. There was no direct involvement of the insular cortex or frontal operculum, and diffusion-weighted imaging did not reveal additional acute ischemic lesions. Therefore, as a hypothesis, the bilateral gustatory disturbance may have resulted from transient compression or dysfunction of adjacent subcortical gustatory pathways, including thalamocortical projections or fibers near the internal capsule, rather than direct putaminal dysfunction alone.

The causal relationship between the putaminal hemorrhage and gustatory dysfunction should be interpreted cautiously. The abrupt onset of complete taste loss, close temporal relationship with the hemorrhage, perihematomal edema extending toward the thalamic region, absence of direct insular or opercular involvement, lack of additional acute ischemic lesions, and gradual recovery support a central stroke-related mechanism. However, the patient also had several potential background contributors to gustatory dysfunction, including mild zinc deficiency, stage 4 chronic kidney disease, smoking and e-cigarette exposure, regular alcohol intake, hypomagnesemia, anemia, and chronic medications. These factors may have increased susceptibility to gustatory dysfunction or contributed to the clinical presentation, although they were unlikely to fully explain the abrupt onset of complete taste loss. Therefore, the putaminal hemorrhage and perihematomal edema were considered the most plausible primary contributors, while recognizing that the gustatory disturbance may have been multifactorial.

Another relevant finding was the old hemorrhagic lesion in the contralateral right putamen. Although the patient had no documented taste disturbance before the current event, pre-stroke gustatory testing was not available. This old contralateral lesion may have increased bilateral vulnerability or reduced compensatory reserve within supratentorial gustatory networks, thereby contributing to the bilateral manifestation after the acute left-sided hemorrhage and perihematomal edema. However, the precise contribution of the old contralateral lesion cannot be determined from this single case. The complete recovery within two months further supports the possibility of reversible functional impairment due to perihematomal edema.

Objective assessment of taste function may be useful in such cases. In the present patient, electrogustometry revealed bilateral impairment with marked asymmetry, despite little subjective awareness of laterality. This discrepancy highlights the value of objective testing for evaluating the distribution and severity of central taste disturbance. However, because gustatory testing is not routinely performed in stroke care, taste disturbance may remain underrecognized, particularly when neurological deficits are mild or when patients are not specifically asked about taste perception.

This study has several limitations. First, this was a single case report with a targeted narrative review, and only a small number of previously reported cases were identified. Therefore, the clinical features, laterality patterns, and prognosis of gustatory disturbance associated with putaminal lesions could not be systematically evaluated. Second, gustatory assessment methods varied among reports, making direct comparison difficult. Third, underreporting and publication bias are likely because taste disturbance may not be systematically assessed in routine stroke care. Fourth, electrogustometry was performed on hospital day 11, after partial subjective improvement had already begun; therefore, objective testing did not capture the initial complete ageusia. Pre-stroke baseline electrogustometry and repeat electrogustometry after clinical recovery were not available, so objective normalization of gustatory function could not be confirmed. Fifth, although the temporal relationship between hemorrhage onset and taste loss supported a central stroke-related mechanism, alternative contributors, including mild zinc deficiency, stage 4 chronic kidney disease, smoking and e-cigarette exposure, alcohol intake, anemia, hypomagnesemia, and background medications, could not be definitively excluded. Finally, the precise anatomical substrate could not be determined, and the contribution of the old contralateral putaminal hemorrhagic lesion remains uncertain.

## Conclusions

Gustatory disturbance after putaminal hemorrhage is rarely reported and may be underrecognized in routine stroke assessment. In the present case, the temporal association and imaging findings suggest, but do not prove, transient involvement of adjacent subcortical gustatory pathways, including thalamocortical projections or fibers near the internal capsule. Targeted inquiry regarding taste perception, combined with objective gustatory testing when available, may improve detection and characterization of this subtle manifestation in patients with deep hemispheric stroke.
